# Activation of nitrogen species mixed with Ar and H_2_S plasma for directly N-doped TMD films synthesis

**DOI:** 10.1038/s41598-022-14233-7

**Published:** 2022-06-20

**Authors:** Jinill Cho, Hyunho Seok, Inkoo Lee, Jaewon Lee, Eungchul Kim, Dougyong Sung, In-Keun Baek, Cheol-Hun Lee, Taesung Kim

**Affiliations:** 1 School of Mechanical Engineering, Sungkyunkwan University, Suwon, 16419 South Korea; 2 SKKU Advanced Institute of Nanotechnology (SAINT), Sungkyunkwan University, Suwon, 16419 South Korea; 3 Samsung Electronic Co. Ltd., Mechatronics R&D Center, 1-1 Samsungjeonja-ro, Hwaseong-si, Gyeonggi-do 18448 South Korea

**Keywords:** Materials science, Nanoscience and technology

## Abstract

Among the transition metal dichalcogenides (TMD), tungsten disulfide (WS_2_) and molybdenum disulfide (MoS_2_) are promising sulfides for replacing noble metals in the hydrogen evolution reaction (HER) owing to their abundance and good catalytic activity. However, the catalytic activity is derived from the edge sites of WS_2_ and MoS_2_, while their basal planes are inert. We propose a novel process for N-doped TMD synthesis for advanced HER using N_2_ + Ar + H_2_S plasma. The high ionization energy of Ar gas enabled nitrogen species activation results in efficient N-doping of TMD (named In situ-MoS_2_ and In situ-WS_2_). In situ-MoS_2_ and WS_2_ were characterized by various techniques (Raman spectroscopy, XPS, HR-TEM, TOF–SIMS, and OES), confirming nanocrystalline and N-doping. The N-doped TMD were used as electrocatalysts for the HER, with overpotentials of 294 mV (In situ-MoS_2_) and 298 mV (In situ-WS_2_) at a current density of 10 mA cm^−2^, which are lower than those of pristine MoS_2_ and WS_2_, respectively. Density functional theory (DFT) calculations were conducted for the hydrogen Gibbs energy (∆G_H_) to investigate the effect of N doping on the HER activity. Mixed gas plasma proposes a facile and novel fabrication process for direct N doping on TMD as a suitable HER electrocatalyst.

## Introduction

Two-dimensional transition metal disulfides (2D-TMD) are the prevailing materials that take advantage of their outstanding physical and electronic properties for various applications such as electronics, sensors, and energy storage devices^1^. Particularly, tungsten disulfide (WS_2_) and molybdenum disulfide (MoS_2_), which belong to the 2D-TMD group, have received considerable attention, especially in the field of hydrogen evolution reaction (HER) electrocatalysts owing to their desirable electrochemical properties^2^. Based on theoretical and empirical research, the S-edges of 2H-MoS_2_ and 2H-WS_2_ with semiconductor properties, play an essential role in the catalytic reaction rather than the inert (0001) basal planes^3,4^. 2H-TMD need to increase the number of active sites that exhibit favorable adsorption of hydrogen ions dissolved in the acidic solution. Several synthesis methods have been introduced to obtain more desirable structures for the HER activity of 2H-TMD materials^5–7^.

In addition to the reported approaches, foreign ion doping into the atomic lattice of MoS_2_ and WS_2_ is an effective way to enhance the HER performance by modifying the electronic properties and conductivity. Several studies have attempted to deal with metal (Sn, Cu, Pd, Co, V, etc.) or non-metal elements (O, P, Te, etc.) to substitute S ions on TMD^8–12^. Those implanted atoms effectively modulate the electronic structure of intrinsic TMD, thereby allowing them to maximize their electrocatalytic performance. Yang et al. elucidated the modified hydrogen binding energy of doped 2H-MoS_2_ depending on group IVA and VA elements by calculating the density functional theory (DFT). This results in an As-doping concentration of 3.125%, making the hydrogen Gibbs energy (∆G_H_) close to zero^13^. Among these dopants, nitrogen (N) doping on TMD has been primarily known in transition nitrides (e.g., Mo_2_N, W_2_N) introduced as metallic materials and even as HER catalysts^14,15^. To incorporate N atoms, remote N_2_ plasma treatment has been most successfully applied to various semiconductors and graphene^16,17^. However, a long processing time is required to perform as an additional doping process after the materials are manufactured. Conversely, the hydrothermal method, where chemical reagents involving nitrogen are added simultaneously, causes environmental problems, and faces low yield^18^. Therefore, it is still challenging to suggest a simple and practical strategy to fabricate N-doped TMD for enhanced HER.

We developed a novel strategy to fabricate wafer-scale N-doped 2H-TMD thin films directly using plasma enhanced-chemical vapor deposition (PE-CVD) at low temperatures. Our previous research on the synthesis of 2H-MoS_2_ and 2H-WS_2_ using Ar and H_2_S plasma has already been reported^19,20^. By extension, 2H-MoS_2_ and 2H-WS_2_, composed of numerous N-doped nanocrystals simultaneously, could be obtained, to prepare an excellent HER electrocatalyst by adding nitrogen gas (N_2_) to Ar/H_2_S plasma (referred to as in situ-MoS_2_ and in situ-WS_2_). In addition, it was observed that the activated N_2_ species, confirmed by in-situ optical emission spectroscopy (OES), was the main factor for inducing the effect of N doping. As a result, both in situ-MoS_2_ and in situ-WS_2_ exhibited enhanced HER activity than pristine TMD, showing overpotentials of 294 and 298 mV at a current density of 10 mA cm^−2^, respectively. To fully understand the HER activity as a function of the implanted N atom, theoretical DFT calculations were carried out to investigate the ∆G_H_ on the basal plane of the as-prepared samples depending on the hydrogen adsorption sites. This confirmed that the lower ∆G_H_ with N atoms in TMD is calculated compared to pristine TMD, enhancing HER performance. Finally, this study demonstrates a unique and facile method for developing advanced electrocatalysts.

## Materials and methods

### PE-CVD system

The PE-CVD system is schematically illustrated in Supplementary Fig. S1. An inductively coupled plasma (ICP) generator operating at 13.56 MHz radio frequency (RF) was used to generate the plasma driven by an electromagnetic field. The 550 W power was applied for synthesizing pristine and N doped TMD thin films. The chamber was evacuated to maintain high vacuum by using a turbo pump. The operating temperature was controlled by the chamber heater located under the substrate.

### Synthesis of pristine TMD thin films

A 4-inch SiO_2_/Si wafer was cleaned to remove organic contaminants by dipping it in ethanol and DI water with sonication. After cleaning, an E-beam evaporator was used to deposit a transition thin metal film (Mo or W) with a thickness of 1 nm on the substrate. Ar/H_2_S plasma (v/v = 1:1) was applied to the thin metal film in the chamber, which had an operating pressure of ~ 10^–6^ Torr and a temperature of 300 °C.

### Synthesis of N doped TMD thin films

The transition thin metal film on the SiO_2_/Si substrate was prepared using the same process. With N_2_ gas flowing at 10 SCCM during Ar/H_2_S plasma, TMD thin films were fabricated, and the N dopants were successfully implanted simultaneously.

### Characterization

The characterization of all samples was performed using Raman spectroscopy, X-ray diffraction (XRD), high-resolution transmission electron microscopy (HR-TEM), X-ray photoelectron spectroscopy (XPS), optical emission spectroscopy (OES), and time of flight secondary ion mass spectrometry (TOF–SIMS) techniques. A Raman microscope (Alpha300 M+, WITec GmbH) was employed with an excitation wavelength of 532 nm. XRD (Smartlab, Rigaku) was used to determine the nanograin size as well as the phase identification of all the samples. HR-TEM (JEM-2100F, JEOL) was utilized to determine the structural configurations of the TMD thin films. Poly-methyl-methacrylate (PMMA) transfer method was applied to prepare TEM samples. Firstly, a PMMA layer was spin cast on the TMD film. Then, a diluted HF solution was used to etch the SiO_2_ layer and separate the PMMA coated TMD films from the substrate. After transferring onto a carbon-coated copper TEM grid, the PMMA layer was dissolved with acetone to remain only TMD films. Cross-sectional TEM images of all samples were also obtained using a focused ion beam (NX2000, Hitachi Ltd.). XPS measurements were conducted to analyze the atomic composition and bonding state using a Thermo Fisher ESCALAB 250 Xi instrument with a Mg Kα X-ray source. The distribution of atoms and molecules in the plasma was investigated using OES (Avantes, Avaspec-2048). The depth profiles of all samples were revealed using TOF-SIMS (TOF-SIMS-5, ION-TOF GmbH).

### Electrochemical measurement

All electrochemical analyses of the samples were conducted using a CHI600D electrochemical workstation comprising a three-electrode system. Pt wire and Ag/AgCl saturated with 4 M KCl were selected as the reference and counter electrodes, respectively. The catalysts were directly synthesized on a carbon glass electrode using a rotating disk electrode (RDE) system (Gamry). This electrode was used as the working electrode. All electrochemical tests were performed under the same conditions at a rotation speed of 1600 rpm in a 0.5 M H_2_SO_4_ electrolyte solution. The reversible hydrogen electrode (RHE) potential from the measured potential was calculated using the following equation:

1 $${\text{E}}_{{{\text{RHE}}}} = {\text{E}}_{{{\text{Ag}} / {\text{AgCl}}}} + 0.{197} + 0.0{\text{59 pH}}$$

The Tafel slopes were calculated to assess the intrinsic HER activity of all samples. The equation below was used to fit linear slopes^21^.

2 $$\eta = {\text{b log}}\left| {\text{j}} \right| + {\text{a}}$$

where η denotes the overpotential, a denotes the exchange current density, and b is the Tafel slope. A 95% IR compensation was applied for all the potentials in the linear sweep voltammetry (LSV) to consider the solution resistance. Electrochemical impedance spectroscopy (EIS) was measured in the frequency range from 100 kHz to 0.01 Hz at the overpotential of − 0.2 V vs RHE.

### Density functional theory (DFT) calculation

All calculations were performed using plane-wave-based DFT, as implemented in the Quantum espresso code^22,23^. Perdew Burke Ernzerhof (PBE) generalized gradient approximation (GGA) was used for the exchange correlation function^24^. The effect of van der Waals interactions was applied using the DFT-D3 method. Supercells (4 × 4 × 1) were designed, and a vacuum space with a thickness of 15 Å was constructed to avoid interactions with other environments in the z direction. An energy cutoff of 40 Ry for the plane wave expansion of the wave functions and a density cutoff of 400 Ry were set after the convergence test. A k-point mesh of 4 × 4 × 1 and a convergence threshold of 10^–6^ eV were adopted. All atomic coordinates in the supercells were relaxed for structural optimization until the Hellmann–Feynman forces were less than 0.01 eV/Å. The hydrogen Gibbs free energies of the pristine and N-doped TMD were calculated using the following equation:

3 $$\Delta {\text{G}}_{{{\text{H}}*}} = \Delta {\text{E}}_{{{\text{H}}*}} - \Delta {\text{E}}_{{{\text{ZPE}}}} - {\text{T}}\Delta {\text{S}}_{{\text{H}}}$$

where ∆E_H*_ is defined as the hydrogen absorption energy on the surface, ∆E_ZPE_ is the zero-energy difference, and ∆S_H_ denotes the entropy difference.

## Results and discussion

**Figure 1:**
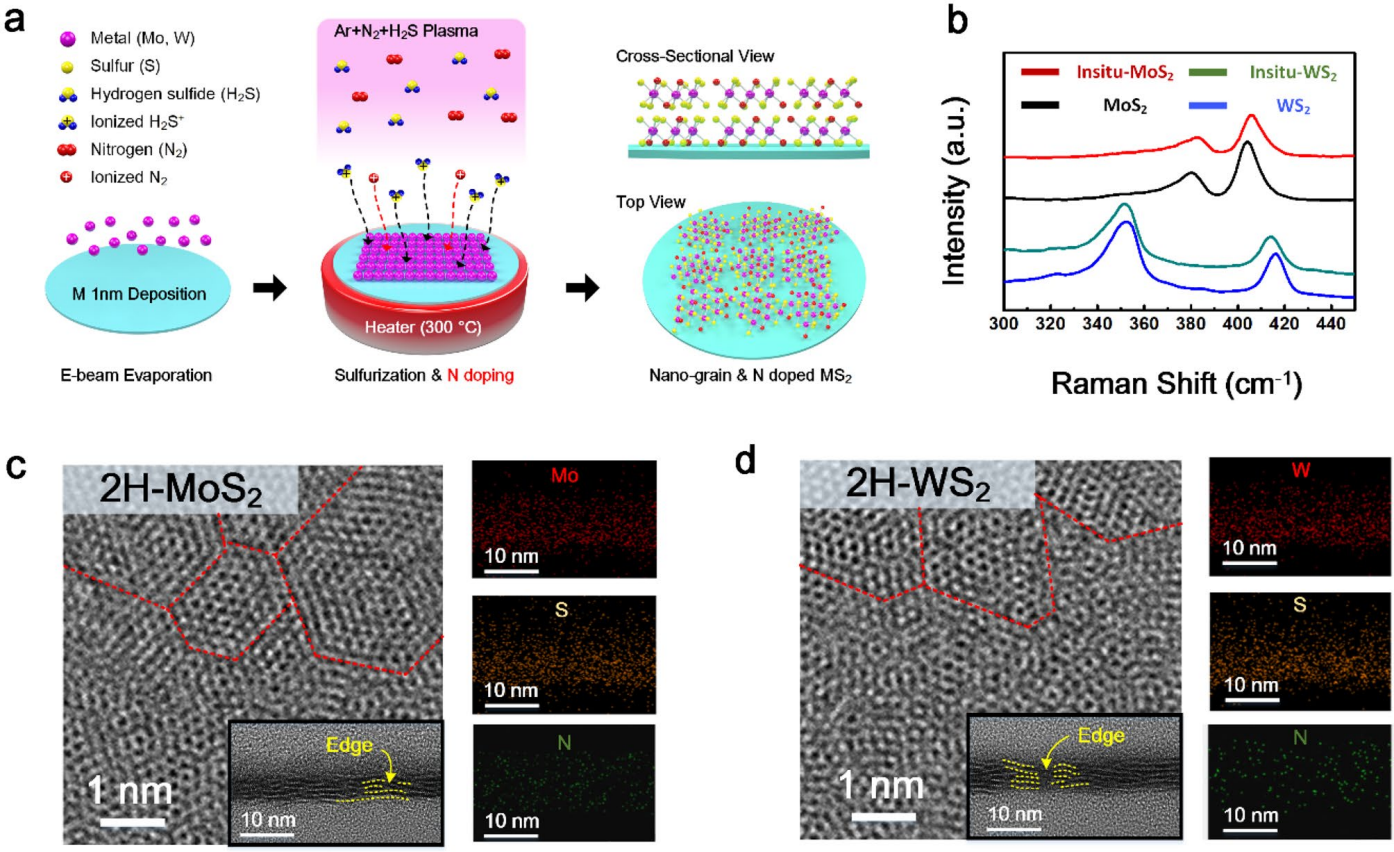
(**a**) Illustration of in situ-doping process during TMD fabrication, (**b**) Raman spectra of pristine TMD and N doped TMD thin films. TEM image of (**c**) in situ-MoS_2_ and (**d**) in situ-WS_2_ samples. Red lines are drawn along with the grain boundary. Inset figures present the cross-sectional TEM images of TMD thin films. EDS images corresponding to each element are arranged vertically on the right side.

An illustration of the in-situ doping process during the synthesis of TMD is depicted in Fig. 1a. To prepare pristine TMD and in situ TMD, Mo or W thin metal films were deposited with a thickness of 1 nm on a 4-in. SiO_2_/Si substrate using E-beam evaporation. Then, the as-prepared metal films were sulfurized using PE-CVD at a temperature of 300 °C for 90 min. The sulfurization process was executed with a mixed gas of Ar and H_2_S (v/v = 1/1) in accordance with a previous study^19,25^. However, a gas mixture containing high-purity N_2_ gas was used to synthesize N-doped TMD thin films. Finally, we obtained a wafer-scale N-doped TMD thin film (Supplementary Fig. S2). Raman spectroscopy measurements were conducted to identify the lattice vibrations of the as-fabricated samples, as shown in Fig. 1b. The two representative bonds of pristine MoS_2_ thin films at 380.8 cm^−1^ and 404.1 cm^−1^ corresponding to in-plane modes (E^1^_2g_) and out-of-plane mode (A_1g_), respectively were discovered. The two major peaks of the pristine WS_2_ thin film at 352.5 cm^−1^ and 416.3 cm^−1^ were also detected in accordance with other reports^26^. Furthermore, while the E^1^_2g_ and A_1g_ peaks for in situ-MoS_2_ blue-shifted to 383.3 cm^−1^ and 405.4 cm^−1^, these peaks of in situ-WS_2_ red shifted to the wavenumber of 351.17 cm^−1^ and 413.8 cm^−1^, respectively, because the charge concentration induced by the introduction of dopants brings about a change in these vibrations, such as compression^27^. Raman mapping of in situ-MoS_2_ and in situ-WS_2_ was investigated to provide spatial distribution of the main peaks corresponding to E^1^_2g_ and A_1g_ mode by using Lorentz filter management (Supplementary Fig. S3). It indicated that the N doped MoS_2_ and WS_2_ were fabricated uniformly through mixed N_2_ + Ar + H_2_S plasma. HR-TEM was carried out to investigate the atomic structural configuration. Compared to the pristine samples, the top-view images of in situ-MoS_2_ and in situ-WS_2_ were confirmed to maintain the intact nanograin hexagonal 2H phase without any cracks, which could be formed by the post-N doping process (Fig. 1c, d)^28^. The ring diffraction in selected area electron diffraction (SAED) pattern indicates that the prepared samples are polycrystalline (Supplementary Fig. S4). It can also be speculated that the grain size did not change even if the TMD thin films involved N doping according to the XRD pattern (Supplementary Fig. S5) analysis. The Scherrer equation can be used to deduce the crystallinity and crystalline size^29^. Basically, the smaller the crystalline size, the broader the diffraction peaks^30^. The broad diffraction peak at 10.4°, corresponding to the (002) plane, implies that the as-fabricated TMD thin films were formed with numerous nano-sized grains, in accordance with the TEM image analysis^31^. Furthermore, the cross-sectional TEM images revealed that the synthesized TMD thin film comprised about 4–5 layers and broken layers, where abundant edges were exposed as active sites. Energy dispersive spectrometer (EDS) mapping images for N-doped TMD indicate that the N atoms were incorporated successfully into the TMD samples.

**Figure 2:**
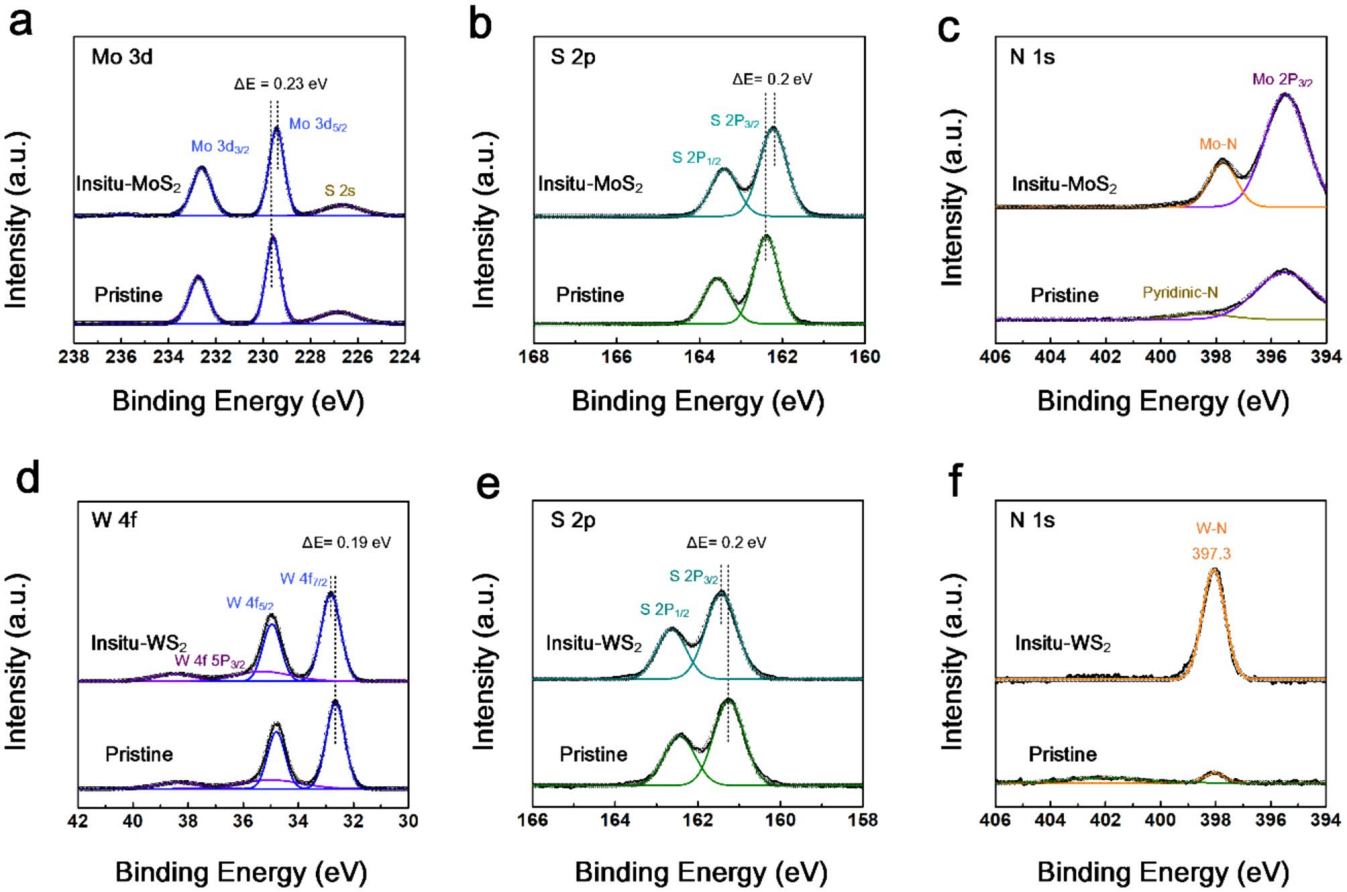
XPS spectra of (**a**) Mo 3d, (**b**) S 2p, and (**c**) N 1 s for pristine MoS_2_ and in situ-MoS_2_. XPS spectra of (**d**) W 4f, (**e**) S 2p, and (**f**) N 1 s for pristine WS_2_ and in situ-WS_2_.

XPS measurements were performed to determine the chemical bonds and components of the samples, as shown in Fig. 2a. Pristine MoS_2_ thin films have the characteristic Mo 3d core level of typical 2H-MoS_2_ at 226.66 eV, 229.43 eV, and 232.59 eV corresponding to the S 2 s, Mo 3d_5/2_, and Mo 3d_3/2_, respectively. In addition, the S 2p core level XPS spectra in Fig. 2b present the major peaks associated with the Mo-S bonding in the lattice of MoS_2_ at 162.38 eV and 163.56 eV. These peaks are consistent with those reported in the literature^32^. However, the additional peak at 398.57 eV in Fig. 2c is assigned to the Mo–N bond that was not detected in pristine MoS_2_^33,34^. Overall, the in situ-MoS_2_ thin film presented a relative peak shift of M 3d and S 2p toward lower binding energies, indicating p-type doping. This indicates that the in-situ process can be regarded as an efficient method to derive N doping on the TMD. This is because nitrogen elements are favorable to combine with Mo rather than sulfur atoms in the lattice of Mo–S^35^. Likewise, Fig. 2d,e also exhibited that pristine WS_2_ has two peaks of W 4f core level at 32.84 eV and 34.89 eV and two peaks of S 2p core level at 161.24 eV and 162.4 eV, corresponding to W 4f_7/2_, W 4f_5/2_, S 3p_3/2_, and S 3p_1/2_, respectively^36^. Unlike the case of n-type doped MoS_2_, the N-doped WS_2_ sample showed the movement of the peak position toward higher binding energy, suggesting n-type doping. In addition, the appearance of a new peak related to the W–N bond at 398.05 eV in Fig. 2f could be observed, implying that N atoms were occupied by replacing S in the lattice of 2H-WS_2_^37^. From this approach, the N doping concentration of in situ MoS_2_ and WS_2_ thin film ran to almost 9.43 at% and 8.3 at%, respectively, while the pristine samples’ doping amount was close to zero percent.

**Figure 3:**
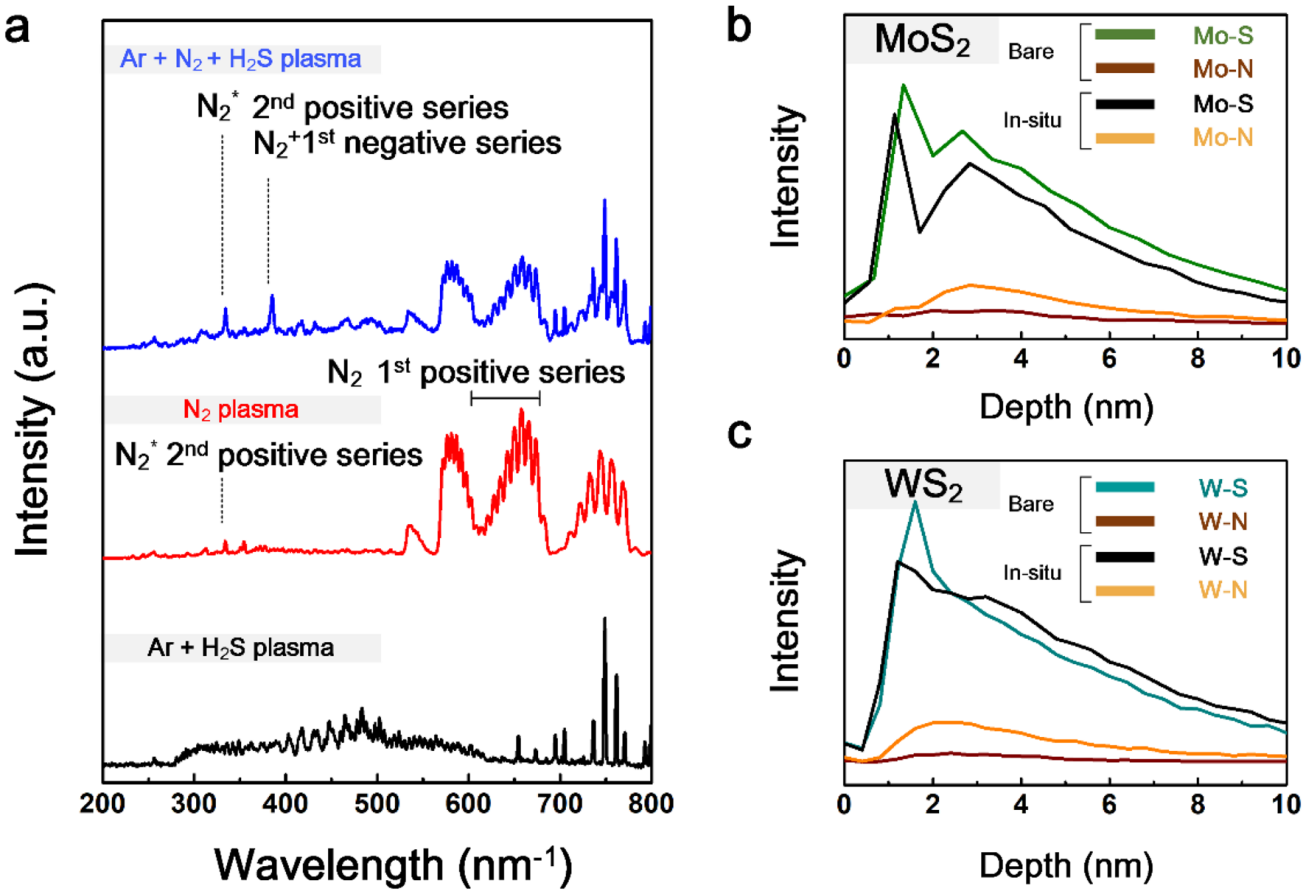
(**a**) OES spectra of three types of plasma depending on the gases. TOF–SIMS analysis of (**b**) MoS_2_ and (**c**) WS_2_ thin films.

These effective N doping attributes to the penning excitation effect by the exitance of argon molecules, allowing TMD to effectively change their atomic composition ratio. In general, the higher ionization energy of Ar in the electric field resulted in the activation of inert N_2_ to generate vigorous 1st negative series N_2_^+^ and 2nd positive series N_2_^*^ as offshoots^38^. These products were confirmed by OES, which is a powerful tool for revealing the excited species during plasma treatment, as shown in Fig. 3a. N_2_^+^ species could only be observed at a wavelength of 427.5 nm^−1^ in the case of mixed-gas plasma, but not for simple N_2_ plasma treatment. In addition, the plasma processes, except for the normal sulfurization treatment, have a distinct peak at 350 nm^−1^ corresponding to the N_2_^*^ species^39^. However, when injecting with Ar, the intensity of N_2_^*^ in the spectrum becomes much stronger than that without Ar. This indicates that activated species play a pivotal role in the N-doping of TMD samples. The importance of the excited N_2_^*^ and charged N_2_^+^ for doping or depositing nitride films at low temperatures has been emphasized previously because of their high reactivity^40–42^. The concentration of the N dopants in the samples treated with only N_2_ plasma is lower than that with facilitating species (Supplementary Fig. S6). Therefore, the generation of active nitrogen species caused by the penning effect in the mixture plasma effectively leads to a favorable combination with TMD. In addition, TOF–SIMS analysis of all samples was conducted to investigate the distribution of the composed atomic bonds with respect to depth, as shown in Fig. 3b,c. The profiles presented the composition for the full range of the as-prepared sample, in which they had a depth of 6–7 nm according to the above-mentioned TEM images. The starting point at 0 nm is the top surface of the sample. It was confirmed that the intensity of the Mo–S bond in the case of the in situ-MoS_2_ thin film decreased, whereas the intensity of the Mo–N bond increased compared to that of pristine MoS_2_. This trend was also observed in the WS_2_ profile. It could be postulated from the increment of the composition associated with the N bonding that the N atom was implanted and combined with the transition metal in the lattice of TMD.

**Figure 4:**
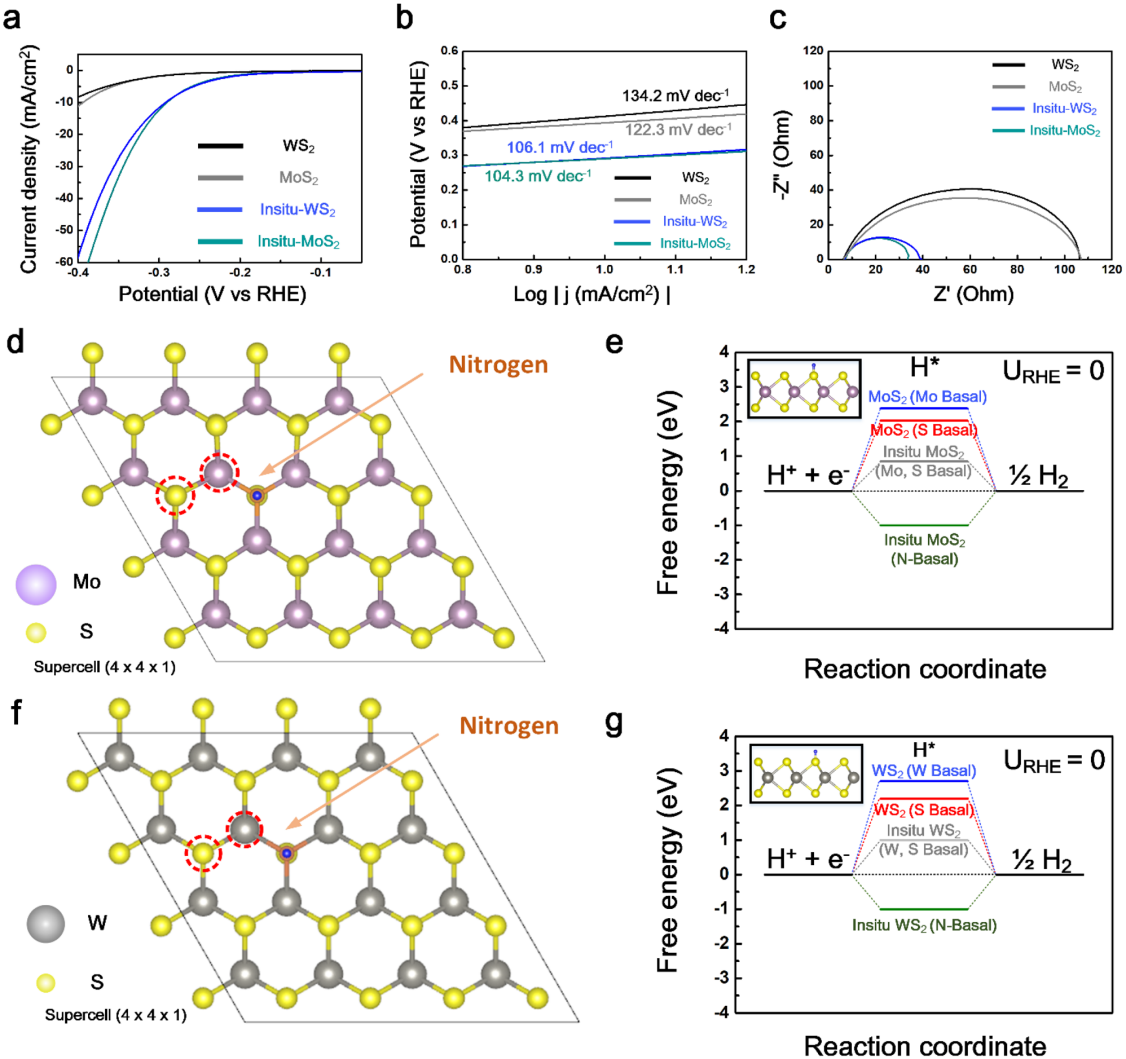
(**a**) LSV curves, (**b**) Tafel plot, and (**c**) EIS for pristine TMD and N doped TMD, respectively, with a scan rate of 5 mVs^−1^ (**d**) A 4 × 4 × 1 in situ-MoS_2_ supercell used for the calculations. (**e**) Hydrogen Gibbs free energy profile for pristine MoS_2_ and in situ-MoS_2_. (**f**) A 4 × 4 × 1 in situ-WS_2_ supercell used for the calculations. (**g**) Hydrogen Gibbs free energy profile for pristine WS_2_ and in situ-WS_2_. The red circle indicates the position of an absorbed hydrogen ion.

LSV was performed to evaluate the HER performance in 0.5 M H_2_SO_4_ solution, as shown in Fig. 4a. The N-doped TMD exhibited better catalytic activity than pristine TMD, resulting in 294 and 298 mV overpotentials at a current density of 10 mA cm^−2^, respectively. In addition, to understand the HER catalytic mechanism, Tafel slopes fitted from the measured LSV curves are presented in Fig. 4b. The lower Tafel slope implies that the electrons can move rapidly to the hydrogen source. In this regard, three principle HER steps on the surface of active materials can be divided depending on the slope value as the reaction rate determinant^43^.

4 $${\text{Volmer}}\;{\text{reaction}}\;\left( {{12}0\;{\text{mV/dec}}} \right){:}\quad {\text{H}}_{{3}} {\text{O}}^{ + } + {\text{e}}^{ - } \to {\text{H}}_{{{\text{ad}}}} + {\text{H}}_{{2}} {\text{O}}$$

5 $${\text{Heyrovsky}}\;{\text{reaction}}\;\left( {{9}0\;{\text{mV/dec}}} \right){:}\quad {\text{H}}_{{{\text{ad}}}} + {\text{H}}_{{3}} {\text{O}}^{ + } + {\text{e}}^{ - } \to {\text{H}}_{{2}} \left( {{\text{gas}}} \right) + {\text{H}}_{{2}} {\text{O}}$$

6 $${\text{Tafel}}\;{\text{reaction}}\;\left( {{3}0\;{\text{mV/dec}}} \right){:}\quad {\text{H}}_{{{\text{ad}}}} + {\text{H}}_{{{\text{ad}}}} \to {\text{H}}_{{2}} \left( {{\text{gas}}} \right)$$

The Tafel slope of pristine MoS_2_ and WS_2_ are 134.2 mV dec^−1^ and 122.3 mV dec^−1^, respectively. However, N doped MoS_2_ and WS_2_ have lower Tafel slopes of 106.1 mV dec^−1^ and 104.3 mV dec^−1^, respectively. Therefore, in this work, the rate-limiting step for the as-prepared samples was the Heyrovsky reaction, following the Volmer reaction to produce H_2_ molecules. EIS measurements were conducted to determine the catalytic kinetics at the interface with an acidic solution, as shown in Fig. 4c. Although the solution electrolyte resistance (Rs) of all samples was the same as 8.1 Ω, the charge transfer resistances (R_ct_) of in situ-MoS_2_ and in situ-WS_2_ were 39 Ω and 35 Ω, respectively, which are much smaller than those of pristine TMD, suggesting enhanced conductivity and electron transferability^44,45^. To estimate the stability of the in situ-MoS_2_ and in situ-WS_2_ samples, an LSV test was performed after 1,000 cycles, as shown in Supplementary Fig. S7. Although there was little degradation after the stability test, it was confirmed to have excellent robustness.

DFT calculations were performed to obtain ∆G_H_ for the reaction intermediate and to analyze the effect of N doping on the TMD catalyst. Figure 4d,f show the 4 × 4 × 1 MoS_2_ and WS_2_ supercell used for the calculations that contain an N atomic concentration of 2.08 at%. ∆G_H_ is the standard descriptor for estimating and predicting the HER catalytic activity^46^. Too positive ∆G_H_ will have difficulty in adsorbing a hydrogen atom on the surface while too negative ∆G_H_ will cause difficulty separating. Hence, the best catalyst should be close to zero. The Gibbs free energy of hydrogen absorption on the basal plane (0001) is over 2 eV, leading to the poor HER performance in Fig. 4e. These results were in good accordance with other reports^47,48^. In contrast, after substituting S with N atoms, it was confirmed that the Mo and S atoms in the direction of the basal plane could be activated with a hydrogen atom by a smaller Gibbs free energy value of approximately 0.85 eV. Likewise, the ∆G_H_ on the basal plane of the in situ-WS_2_ was lowered from 2.1 to 1.0 eV in Fig. 4g. It can be concluded from the calculated Gibbs free energy that the introduction of N atoms in the 2H-TMD stimulates catalytic activation with hydrogen atoms.

## Conclusion

Plasma-assisted sulfurization in a mixture of N_2_ + Ar + H_2_S environment demonstrated that N-doping TMD thin films were synthesized in one step by confirming the Raman spectra and XPS. OES spectra analysis revealed the role of N_2_^+^ species in deriving the high N-doping concentration during the synthesis of TMD. In particular, N_2_^+^ ions were discovered only in the presence of Ar gas, which has sufficient ion energy to bring about the penning effect. This activated species was ascribed to the fabrication of N-doped TMD as efficient HER catalysts. The as-synthesized in situ-MoS_2_ and WS_2_ exhibited increased catalytic activity, resulting in overpotentials of 294 and 298 mV at a current density of 10 mA cm^−2^, respectively. In addition, DFT calculations supported that incorporating N atoms on the TMD could have lower hydrogen Gibbs free energy than pristine TMD, especially on the basal plane.

## Supplementary Information

Supplementary Figures.

## Data Availability

All relevant data are within the paper.
