# Supplementary material for: Activation of nitrogen species mixed with Ar and H2S plasma for directly N-doped TMD films synthesis
Source: Sci Rep. 2022 Jun 20;12:10335. doi: 10.1038/s41598-022-14233-7 (PMC9209500; doi:10.1038/s41598-022-14233-7)
Supplement: Supplementary file 1 — Supplementary Figures. [file 41598_2022_14233_MOESM1_ESM.docx]

Supplementary Information

Activation of Nitrogen Species Mixed with Ar and H_2_S Plasma for directly N-doped TMD Films Synthesis

**Jinill Cho^1,^**^+^**, Hyunho Seok^2,^**^+^**, Inkoo Lee^1,^**^+^**, Jaewon Lee^1^, Eungchul Kim^1^, Dougyong Sung^3^, In-Keun Baek^3^, Cheol-Hun Lee^3^, and Taesung Kim^1,2,*^**

1 Sungkyunkwan University, School of Mechanical Engineering, Suwon, 16419, South Korea.

2 Sungkyunkwan University, SKKU Advanced Institute of Nanotechnology (SAINT), Suwon, 16419, South Korea.

^3^ Samsung Electronic Co., Ltd, Mechatronics R&D Center, 1-1 Samsungjeonja-ro, Hwaseong-si, Gyeonggi-do 18448, South Korea

* Corresponding author: tkim@skku.edu

^+^ These authors contributed equally to this work.


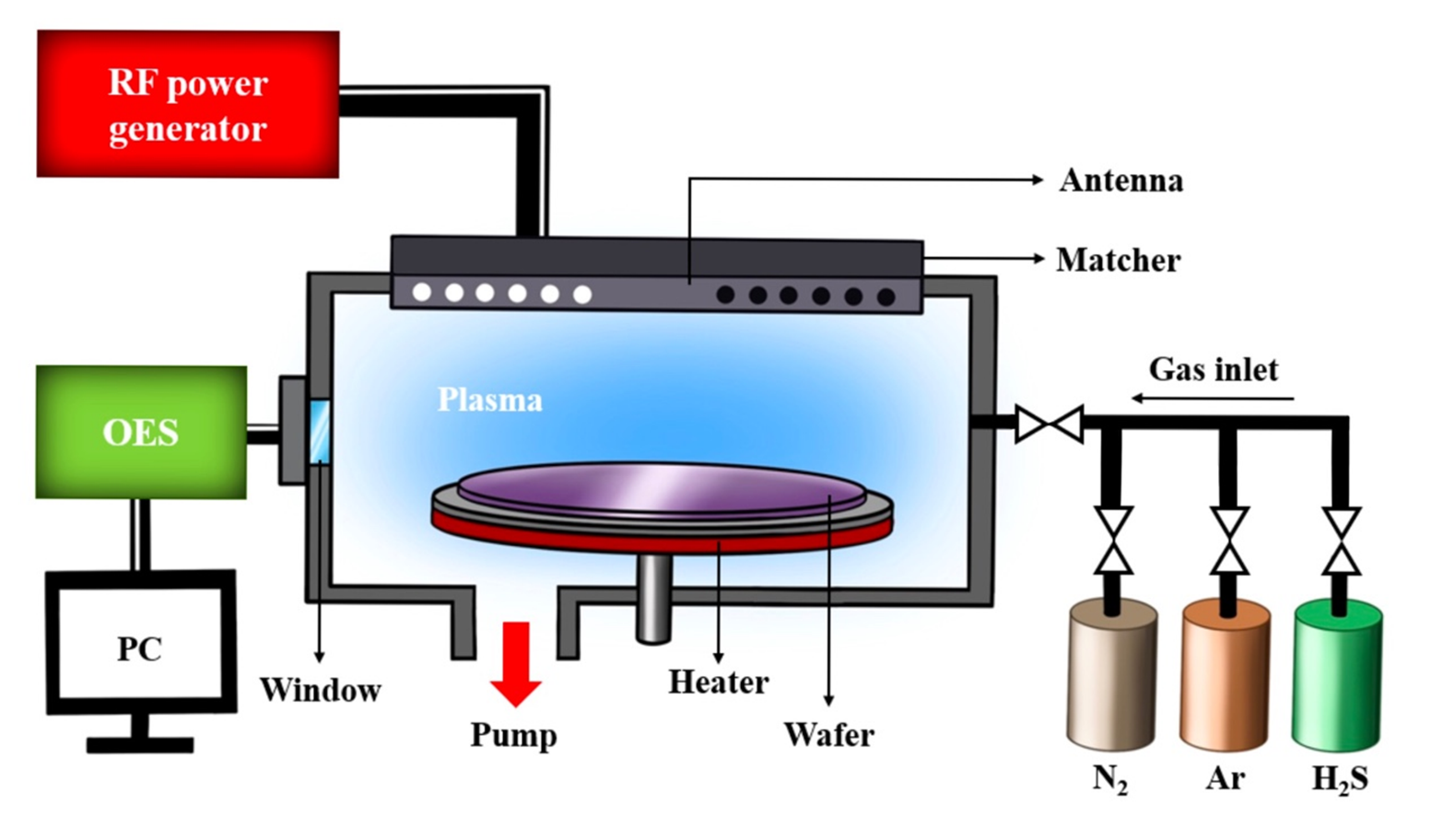


**Figure S1.** Schematic diagram of PE-CVD system.

**
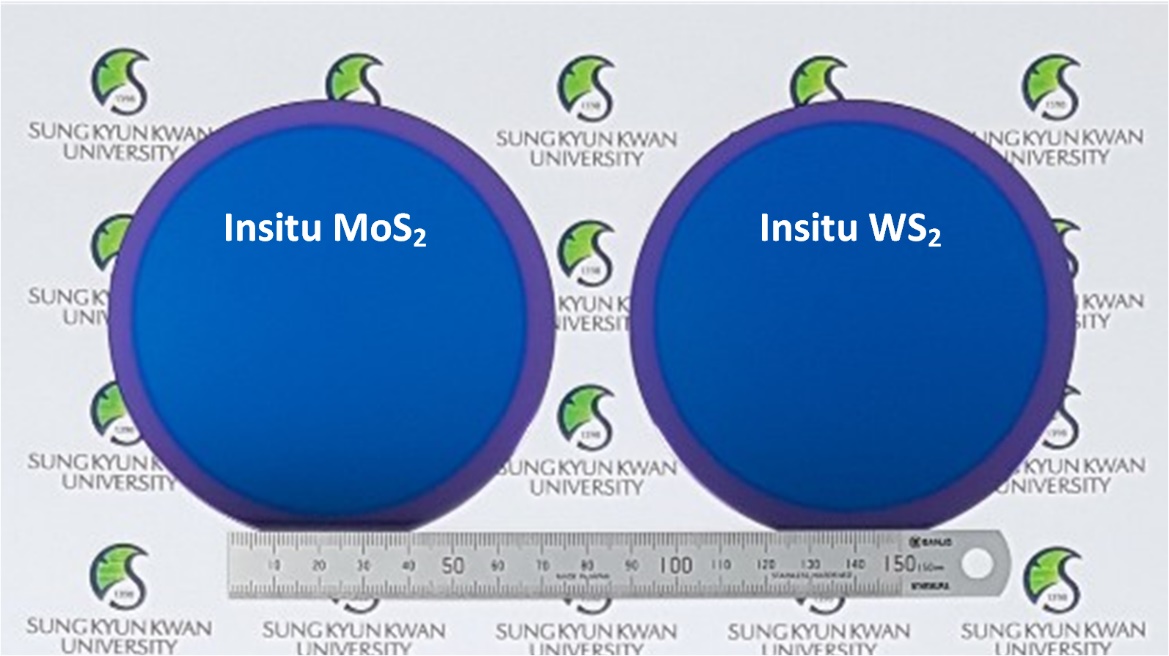
**

**Figure S2.** The photograph of in situ-MoS_2_ and in situ-WS_2_ samples.


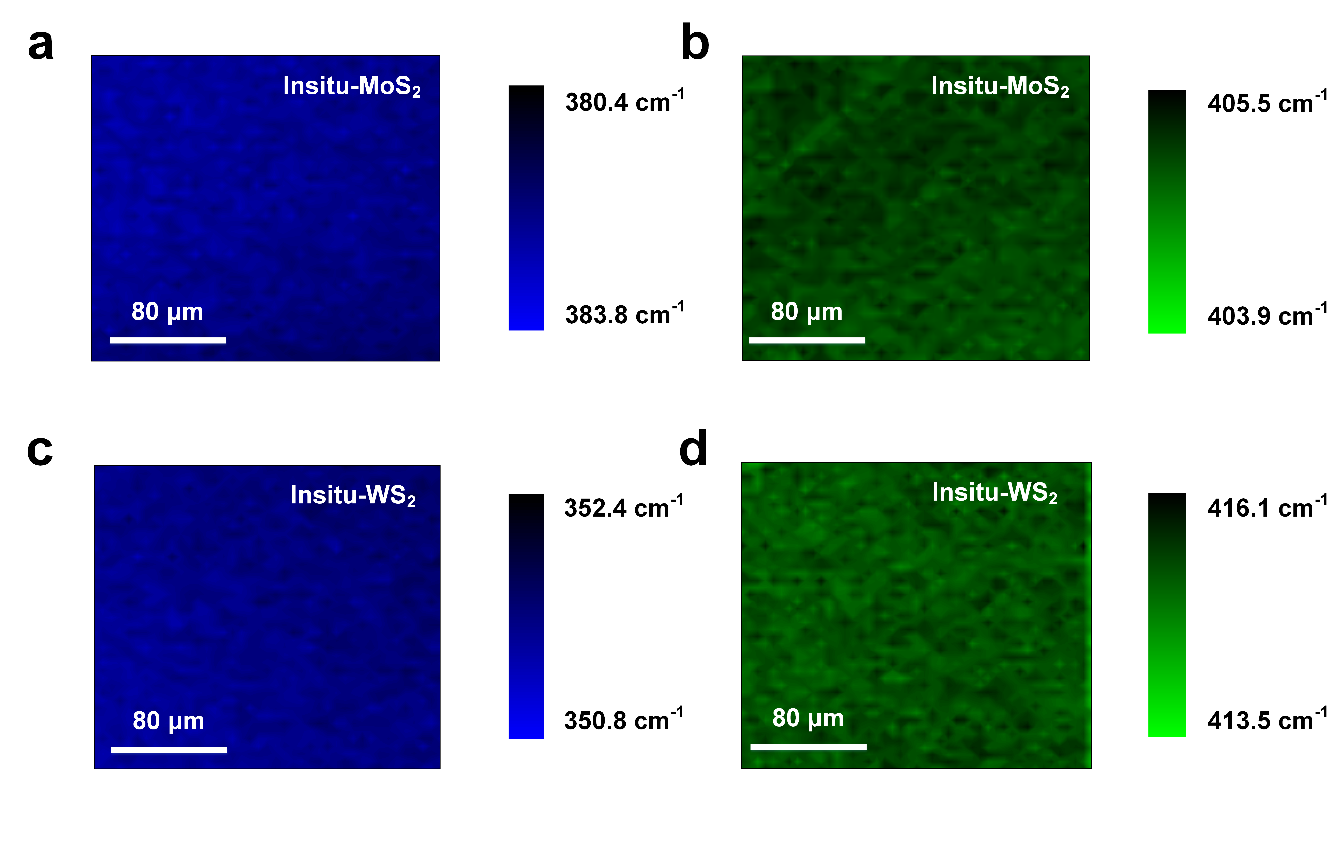


**Figure S3.** Raman mapping of in situ-MoS_2_ and in situ-WS_2_ corresponding to (a,c) in-plane mode (E^1^_2g_) and (b,d) out-of-plane mode (A_1g_).


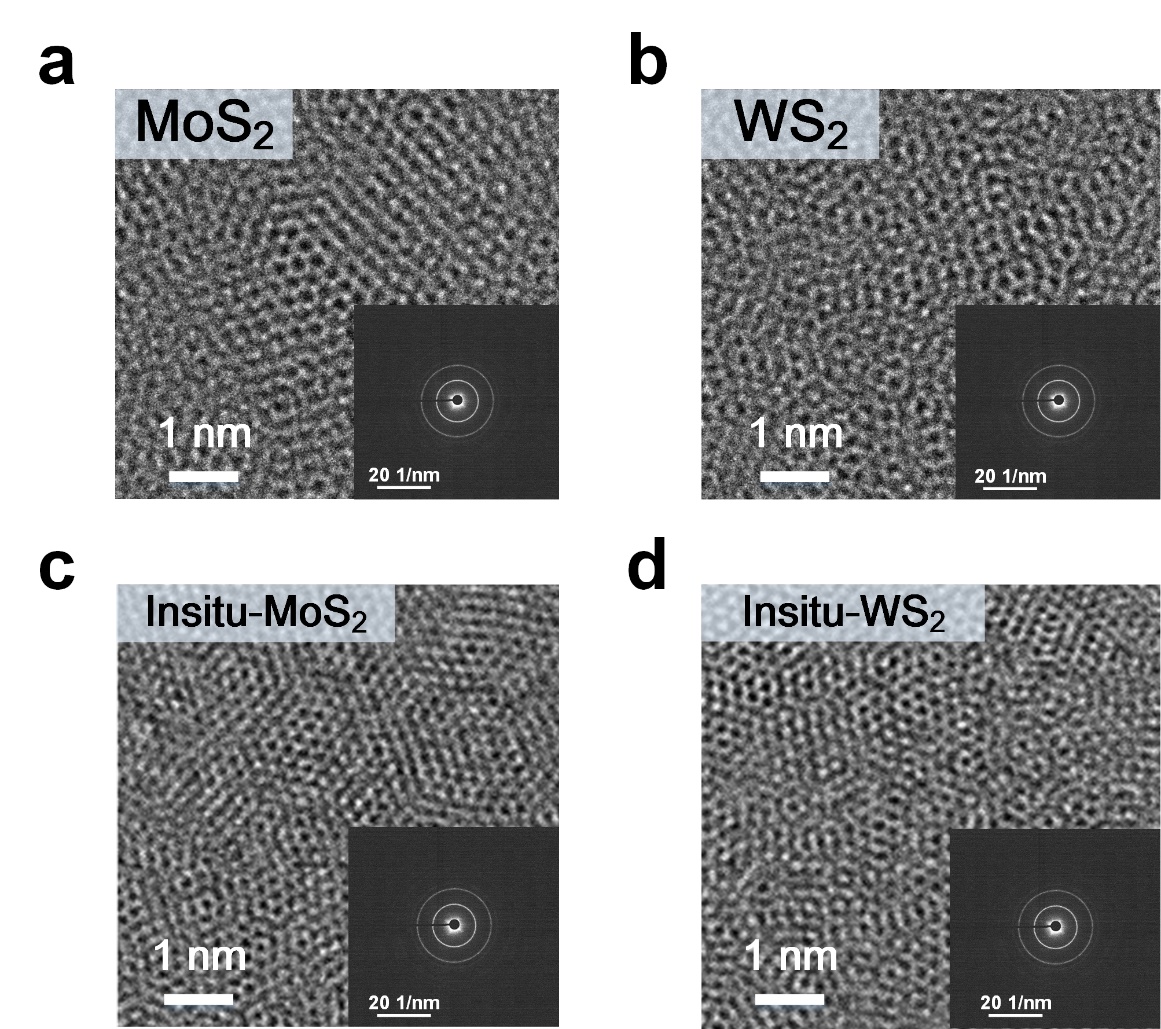


**Figure S4.** TEM image of (a) pristine MoS_2_, (b) pristine WS_2_, (c) in situ-MoS_2_, and (d) in situ-WS_2_ samples. Inset figures indicate SAED patterns.


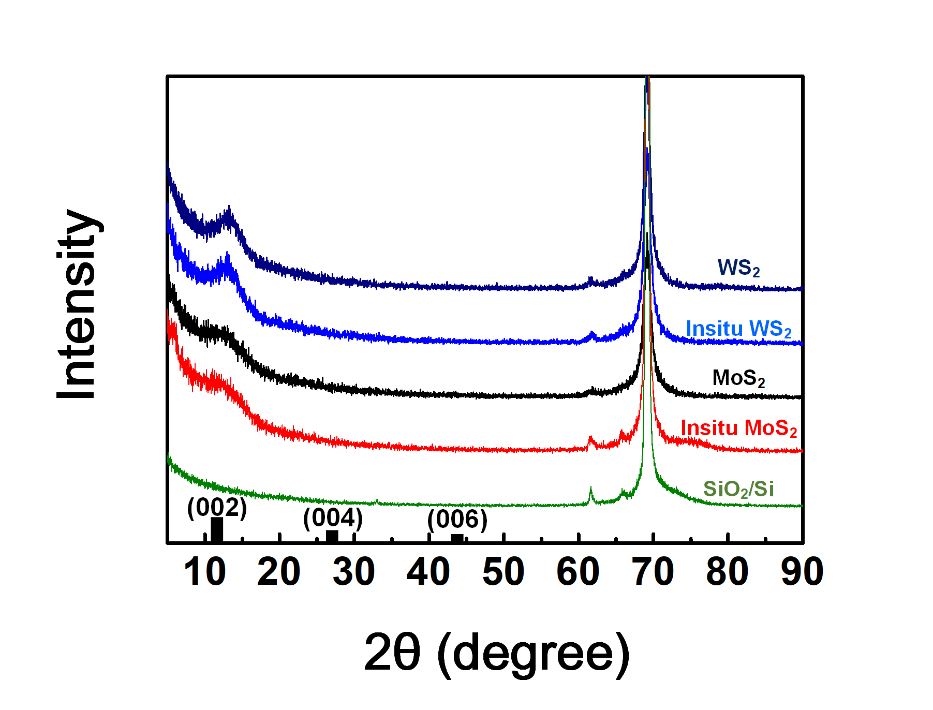


**Figure S5.** XRD pattern of all samples.


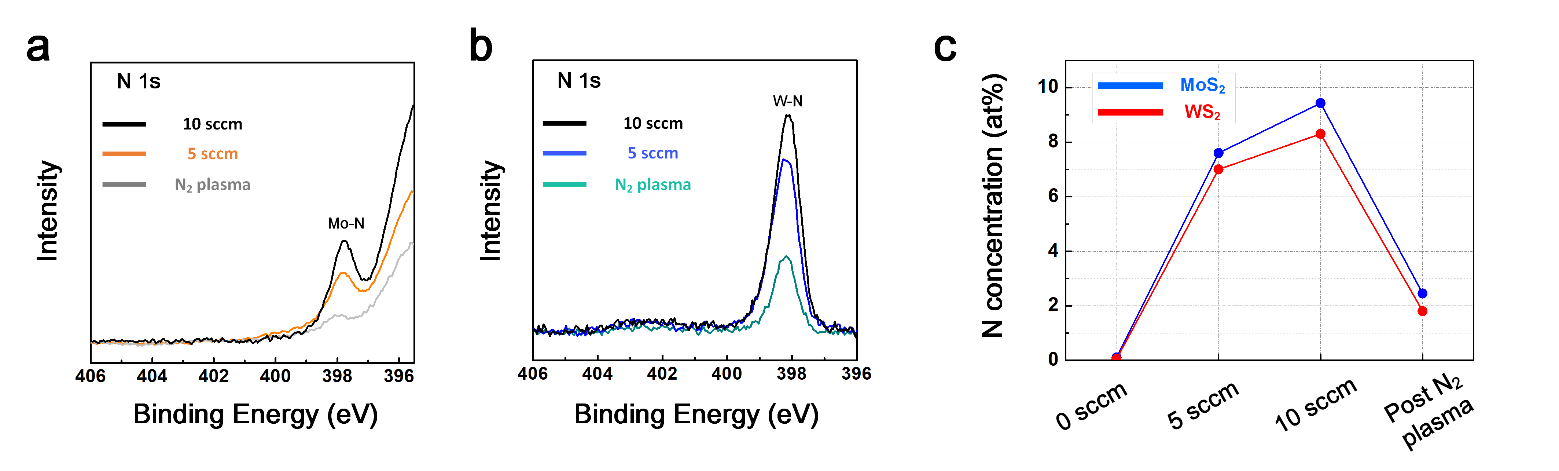


**Figure S6.** XPS spectra of N 1s core level of (a) MoS_2_ and (b) WS_2_ depending on the gas flow rate. The spectra of post N_2_ plasma treated MoS_2_ and WS_2_ is also presented in the graph. (c) N dopants concentration analyzed from XPS data.

**
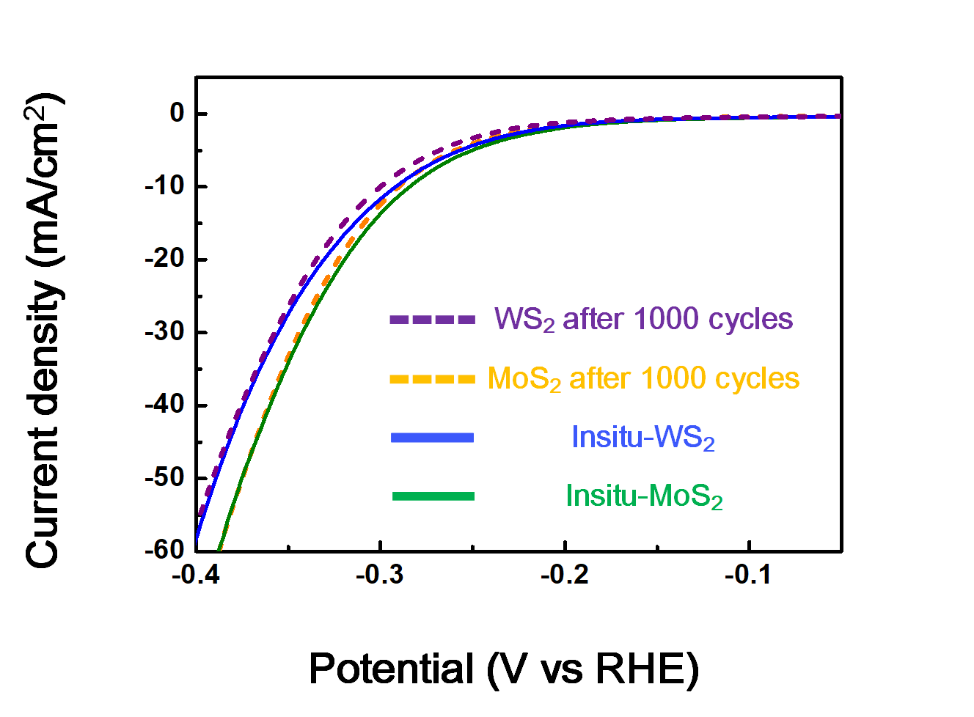
**

**Figure S7.** LSV curves of in situ-MoS_2_ and in situ-WS_2_ before and after 1000 cycles.
